# Psychological Support in Times of Crisis: The Experience of The Al Haouz Earthquake Helpline in Morocco

**DOI:** 10.1192/j.eurpsy.2026.11338

**Published:** 2026-07-31

**Authors:** S. Boughdadi, R. Hayat, I. Adali, F. Manoudi

**Affiliations:** Psychiatry, Mohammed VI university hospital, Marrakech, Morocco

## Abstract

**Introduction:**

On September 8, 2023, a 6.9-magnitude earthquake struck the Al Haouz region, causing significant casualties and psychological trauma. In response, the Psychiatry Department of the Mohammed VI University Hospital in Marrakech established a psychological support helpline.

**Objectives:**

This work aims to describe the epidemiological data collected from the crisis helpline that was put in place in response to the earthquake in Morocco in 2023

**Methods:**

This is a retrospective descriptive study analyzing epidemiological and clinical data collected over the first two months of the helpline’s operation. The service was available for adults affected by the earthquake within the Marrakech-Safi region.

**Results:**

A total of 113 individuals were included. The last earthquake-related call was received on September 27, 2023 (day 19 post-event). Women constituted 78% of callers, with an average age of 35 years (range: 17–80). Most calls originated from Marrakech (76.1%), followed by the Al Haouz region (13.2%). Clinically, 75.2% had no prior psychiatric history. The most common symptoms were anxiety (79.6%), sleep disturbances (71.7%), and mood symptoms such as sadness (11.5%). Decreased appetite (8.8%), somatic complaints (7%), and dissociative or intrusive symptoms (6.2%) were also reported. All callers received psychological first aid, including active listening, psychoeducation, and practical advice (media restriction, sleep hygiene, phytotherapy). Emergency psychiatric consultation was recommended in 12.4% of cases, and deferred consultation in 22.1%.

**Conclusions:**

This experience highlights the crucial need for psychological support services following natural disasters. While immediate benefits were evident, further research is required to evaluate the long-term preventive impact of such interventions.

**Disclosure of Interest:**

None Declared

